# Medical students’ perceptions of a career in family medicine

**DOI:** 10.1186/s13584-017-0193-9

**Published:** 2018-02-12

**Authors:** Sody Naimer, Yan Press, Charles Weissman, Rachel Yaffa Zisk-Rony, Yoram G. Weiss, Howard Tandeter

**Affiliations:** 1 Department of Family Medicine, and Siaal Research Center for Family Medicine and Primary Care, Faculty of Health Sciences, Ben-Gurion University of the Negev, Beer- POB 653, 84105 Beer-Sheva, Israel; 2 Department of Anesthesiology and Critical Care Medicine. Hadassah-Hebrew University Medical Center, Hebrew University – Hadassah School of Medicine, Jerusalem, Israel; 3 Hebrew University – Hadassah Henrietta Szold School of Nursing, Jerusalem, Israel

**Keywords:** Family medicine, Medical education, Medical students, Career choice, Medical specialty

## Abstract

**Background:**

In Israel, there is a shortage of family medicine (FM) specialists that is occasioned by a shortage of students pursuing a FM career.

**Methods:**

A questionnaire, based on methods adapted from marketing research, was used to provide insight into the medical specialty selection process. It was distributed to 6^th^-year medical students from two Israeli medical schools.

**Results:**

A response rate of 66% resulted in collecting 218 completed questionnaires. Nineteen of the students reported that they were interested in FM, 68% of them were women. When compared to students not interested in FM, the selection criteria of students interested in FM reflected greater interest in a bedside specialty which provides direct long-term patient care. These latter students were also more interested in a controllable lifestyle that allowed time to be with family and children and working outside the hospital especially during the daytime. These selection criteria aligned with their perceptions of FM, which they perceived as providing them with a controllable lifestyle, allowing them to work limited hours with time for family and having a reasonable income to lifestyle ratio. The students not interested in FM, agreed with those interested in FM, that the specialty affords a controllable lifestyle and the ability to work limited hours Yet, students not interested in FM more often perceived FM as being a boring specialty and less often perceived it as providing a reasonable income to lifestyle ratio. Additionally, students not interested in FM rated the selection criteria, academic opportunities and a prestigious specialty, more highly than did students interested in FM. However, they perceived FM as neither being prestigious nor as affording academic opportunities

**Conclusion:**

This study enriches our understanding of the younger generation's attitudes towards FM and thus provides administrators, department chairs and residency program directors with objective information regarding selection criteria and the students’ perceptions of FM. We identified the disconnect between the selection criteria profiles and the perceptions of FM of students not inclined to pursue a residency in FM. This allowed for recommendations on how to possibly make FM more attractive to some of these students.

## Background

*“Rabbi [Judah HaNassi] said: Which is the right path for man to choose for himself? Whatever is harmonious for the one who does it, and harmonious for mankind”* [1].

Choosing a specialty is the major decision students make during their medical school years. This decision must take into consideration personal issues such as lifestyle, desired professional fulfilment and personal self-satisfaction. In many countries, there is a significant discrepancy between the needs of the healthcare system for primary care physicians and the number of students interested in a primary care career, leading to shortages of primary care specialists [2]. Despite the fact that effective and sufficient primary care is associated with improved health outcomes [3], data from Israel revealed that 54% of the physician workforce was employed in medical centers as opposed to 39% in the community [4]. The failure to meet the demand for primary care is established and continues to deepen in Israel, although an updated formal analysis has not been performed recently. This problem is not unique to Israel, generalists make up only about 30 % of all physicians in OECD (Organization for Economic Cooperation and Development) countries [5], although proportions vary, from 50% in Australia and Canada, to 30 % in the United Kingdom, to 12 % in the United States (this figure rises to 30% if general internists and general pediatricians are included [6]). Therefore, in many countries, these low percentages translate into inadequate numbers of primary care physicians causing an inability to meet the needs of the population. For example, of the nearly 956 million visits that Americans made to office-based physicians in 2008, 51% were to primary care physicians [7]. A survey from the United States, showed that the proportion of graduates choosing a primary care specialty dropped from 61% in 1997 to 42% in 2006 [8]. Similarly, between 2005 and 2009, only 28% of medical school graduates in the United Kingdom planned to go into general practice [9].

Currently, there is no major shortage of primary care physicians in the center of the country and in the large cities, However, there is a shortage in the peripheral areas of the country which are often rural. As a result, in 2015, the Israel Ministry of Health included family medicine residents willing to train in peripheral areas in the incentive program included in the physicians' union contract of 2011 to entice physicians to moving to the periphery. Furthermore, the primary care physician population is aging as many physicians who emigrated from the former Soviet Union in the 1990's reach retirement age. This situation coupled with a growing and aging population that has longer life spans potends a impending shortage. Therefore, it is important to examine ways to attract additional students to the specialty. This study thus aims to delineate Israeli medical students' perceptions of FM and how these perceptions correlate with the relative importance of various selection criteria. This involved using methods adapted from marketing research which is detailed in the Methods Section [10]. It is important to clarify these issues in order to develop strategies to attract more students to careers in FM and thus avoid future workforce shortages.

## Methods

### Selection of study subjects

Data were collected from final-year medical students (6^th^ year which is the final year before internship) of two Israeli medical schools using a questionnaire designed to elucidate the various aspects of choosing a specialty by medical students. The questionnaire was distributed to three successive classes of final-year students at the Hebrew University, Hadassah School of Medicine, Jerusalem, Israel, plus one class at the Ben-Gurion University of the Negev School of Medicine, Beer-Sheva, Israel.

### Study design

The methodological concept was adapted from marketing research and hypothesizes that when a consumer’s (i.e. student's) criteria match their perceptions of a product’s (i.e. specialty) features, the likelihood of a purchase (selecting the specialty) increases [10]. To provide insights into the selection process, this methodology examined both sides of the marketing equation, i.e. the students’ selection criteria and their perceptions of the various specialties.

### Measurements

The design was based on the AIUAPR (awareness, interest, understanding, attitudes, purchase and repeat purchase) and other models of consumer behavior) [10–12]. The questionnaire queried the students on the following:

1. Interest of the students in each of 19 medical specialties.
2. Importance of each of 25 criteria on the students’ choice of a medical specialty.
3. Perceptions (16 items) of 6 key specialties: pediatrics, orthopedic surgery, anesthesiology, obstetrics/gynecology, general surgery, and FM.
4. Level of consideration in pursuing a career in each of these specialties.
5. Demographic data.

A 5-point Likert scale was used for answering the questions in Sections 1-4. Results from the current dataset have been published without an in-depth focus on issues surrounding FM [10, 13–15].

The study received approval from the Institutional Review Board of the Hadassah Medical Organization. Participation was purely voluntary and there were no incentives aside from the intention to assist the researchers and contribute to the study. Questionnaires were completely anonymous and, therefore, the sampling design precluded assessment of the responder versus non-responder characteristics.

### Statistical analysis

The data collected from the questionnaires were entered into Excel 2003 (Microsoft Inc., Redmond, WA) spreadsheets and then underwent statistical analysis with Systat Version 12 (Systat Software Inc., San Jose, CA).

Chi-square analysis was performed for binomial responses while categorical data were presented as frequency distributions. Two-tailed Student *t* tests compared continuous variables, with Bonferroni corrections used for multiple comparisons.

For statistical analysis, the Likert Scale was treated as a quantitative expression of qualitative data. When reported as categorical data, the 5-points of the Likert Scale were reduced to three categories (the percentages of the responses from the two points representing negative responses were added together as were the percentages of two points representing positive responses plus the middle point). The percentage of responses for each of the three categories was calculated. Statistical significance was considered as a *p*<0.05.

## Results

A response rate of 66% resulted in collecting the views of 218 6^th^-year medical students. Forty-one (19%) reported that they were interested in FM. Female students were significantly more interested in FM when compared to male students (68% vs. 32%; *P*=0.025). Although 54% of those interested in FM were married as opposed to only 44% of those interested in other specialties, the difference was not statistically significant (Table 1). No differences were found between students in the two medical schools.

**Table 1:** Sociodemographic characteristics

	ALL Fam Med (*N*=41)		ALL Others (*N*=177)		*p* value
	N	%	N	%	
Age					
21-23	1	2.4%	8	4.5%	0.948
24-26	15	36.6%	70	39.8%	
27-29	16	39.0%	64	36.4%	
30-32	7	17.1%	28	15.9%	
32+	2	4.9%	6	3.4%	
	41		176		
Gender					
Female	28	68.3%	86	48.6%	0.025
Male	13	31.7%	91	51.4%	
	41		177		
Family status					
Single	18	43.9%	100	56.5%	0.343
Married	22	53.7%	74	41.8%	
Widow	1	2.4%	3	1.7%	
	41		177		

**Fig. 1:**
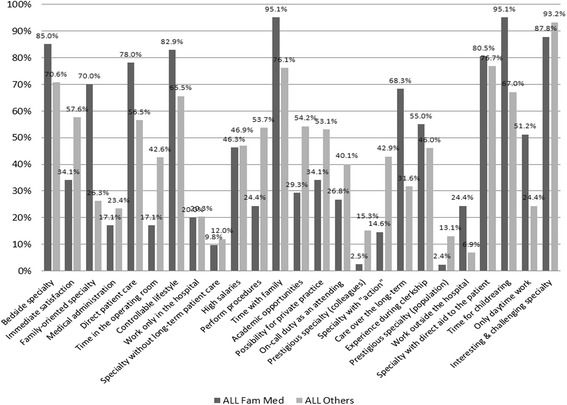
Importance of Selection Criteria - Final Year Medical Students Interested in Family Medicine vs those Interested in Other Specialties

When compared to students not interested in FM, these students' selection criteria reflected greater interest in a bedside specialty which provides direct long-term patient care (Fig. 1 and Table 2). These FM oriented students were also more interested in a controllable lifestyle that allowed time to be with family and children. This lifestyle orientation was further demonstrated by their interest in work outside the hospital especially during the daytime. These selection criteria aligned with their perceptions of FM, which they perceived as providing them with a controllable lifestyle, allowing them to work limited hours with time for family and having a reasonable income to lifestyle ratio (Table 3).

**Table 2:** Importance of Specialty Selection Criteria: Comparison of Students Interested in FM vs those Interested in Other Specialties

	ALL Fam Med (*N*=41)			ALL Others (*N*=177)			*p* value	MALES Fam Med (*N*=13)			MALES Others (*N*=91)			*p* value	FEMALE Fam Med (*N*=28)			FEMALE Others (*N*=86)			*p* value
	N	%	mis	N	%	mis		N	%	mis	N	%	mis		N	%	mis	N	%	mis	
Bedside specialty	34	85.0%	1	125	70.6%	0	0.075	12	100.0%	1	58	63.7%	0	0.008	22	78.6%	0	67	77.9%	0	1.000
Immediate satisfaction	14	34.1%	0	102	57.6%	0	0.009	6	46.2%	0	55	60.4%	0	0.376	8	28.6%	0	47	54.7%	0	0.018
Family-oriented specialty	28	70.0%	1	46	26.3%	2	<0.0001	7	58.3%	1	18	20.0%	1	0.008	21	75.0%	0	28	32.9%	1	<0.0001
Medical administration	7	17.1%	0	41	23.4%	2	0.531	2	15.4%	0	25	27.5%	0	0.506	5	17.9%	0	16	19.0%	2	1.000
Direct patient care	32	78.0%	0	100	56.5%	0	0.013	9	69.2%	0	43	47.3%	0	0.235	23	82.1%	0	57	66.3%	0	0.154
Time in the operating room	7	17.1%	0	75	42.6%	1	0.002	2	15.4%	0	41	45.6%	1	0.068	5	17.9%	0	34	39.5%	0	0.041
Controllable lifestyle	34	82.9%	0	116	65.5%	0	0.039	11	84.6%	0	56	61.5%	0	0.130	23	82.1%	0	60	69.8%	0	0.231
Work only in the hospital	8	20.0%	1	36	20.3%	0	1.000	1	8.3%	1	16	17.6%	0	0.685	7	25.0%	0	20	23.3%	0	1.000
Specialty without long-term patient care	4	9.8%	0	21	12.0%	2	0.793	0	0.0%	0	11	12.2%	1	0.351	4	14.3%	0	10	11.8%	1	0.745
High salaries	19	46.3%	0	83	46.9%	0	1.000	8	61.5%	0	46	50.5%	0	0.559	11	39.3%	0	37	43.0%	0	0.827
Perform procedures	10	24.4%	0	95	53.7%	0	0.001	4	30.8%	0	56	61.5%	0	0.068	6	21.4%	0	39	45.3%	0	0.027
Time with family	39	95.1%	0	134	76.1%	1	0.005	12	92.3%	0	61	67.8%	1	0.102	27	96.4%	0	73	84.9%	0	0.182
Academic opportunities	12	29.3%	0	96	54.2%	0	0.005	2	15.4%	0	55	60.4%	0	0.003	10	35.7%	0	41	47.7%	0	0.285
Possibility for private practice	14	34.1%	0	94	53.1%	0	0.037	3	23.1%	0	59	64.8%	0	0.006	11	39.3%	0	35	40.7%	0	1.000
On-call duty as an attending	11	26.8%	0	71	40.1%	0	0.152	5	38.5%	0	30	33.0%	0	0.757	6	21.4%	0	41	47.7%	0	0.016
Prestigious specialty (colleagues)	1	2.5%	1	27	15.3%	0	0.034	0	0.0%	1	20	22.0%	0	0.117	1	3.6%	0	7	8.1%	0	0.677
Specialty with "action"	6	14.6%	0	76	42.9%	0	0.001	4	30.8%	0	46	50.5%	0	0.240	2	7.1%	0	30	34.9%	0	0.004
Care over the long-term	28	68.3%	0	56	31.6%	0	<0.0001	9	69.2%	0	22	24.2%	0	0.002	19	67.9%	0	34	39.5%	0	0.016
Experience during clerkship	22	55.0%	1	81	46.0%	1	0.381	6	50.0%	1	39	42.9%	0	0.760	16	57.1%	0	42	49.4%	1	0.519
Prestigious specialty (population)	1	2.4%	0	23	13.1%	1	0.054	0	0.0%	0	17	18.9%	1	0.119	1	3.6%	0	6	7.1%	1	0.679
Work outside the hospital	10	24.4%	0	12	6.9%	2	0.002	1	7.7%	0	5	5.5%	0	0.561	9	32.1%	0	7	8.3%	2	0.004
Specialty with direct aid to the patient	33	80.5%	0	135	76.7%	1	0.683	11	84.6%	0	69	75.8%	0	0.728	22	78.6%	0	66	77.6%	1	1.000
Time for childrearing	39	95.1%	0	118	67.0%	1	<0.0001	12	92.3%	0	50	54.9%	0	0.013	27	96.4%	0	68	80.0%	1	0.041
Only daytime work	21	51.2%	0	43	24.4%	1	0.001	4	30.8%	0	15	16.5%	0	0.250	17	60.7%	0	28	32.9%	1	0.014
Interesting & challenging specialty	36	87.8%	0	164	93.2%	1	0.328	12	92.3%	0	85	93.4%	0	1.000	24	85.7%	0	79	92.9%	1	0.261

**Table 3:** Medical Students’ Perceptions of FM: Comparison of Students Interested in FM vs those Interested in Other Specialties

	ALL Fam Med (*N*=41)			ALL Others (*N*=177)			*p* value	MALES Fam Med (*N*=13)			MALES Others (*N*=91)			*p* value	FEMALE Fam Med (*N*=28)			FEMALE Others (*N*=86)			*p* value
Perceptions	N	%	mis	N	%	mis		N	%	mis	N	%	mis		N	%	mis	N	%	mis	
Advanced Specialty	12	29.3%	-	36	20.7%	3	0.296	4	30.8%	0	18	20.0%	1	0.468	8	28.6%	0	18	21.4%	2	0.447
Interesting Specialty	28	68.3%	-	25	14.2%	1	<0.0001	11	84.6%	0	10	11.0%	0	<0.0001	17	60.7%	0	15	17.6%	1	<0.0001
Boring Specialty	7	17.9%	2	96	54.5%	1	<0.0001	0	0.0%	0	54	60.0%	1	<0.0001	7	25.9%	1	42	48.8%	0	0.046
Stressful Specialty	5	12.2%	-	24	13.7%	2	1.000	3	25.0%	1	10	11.1%	1	0.211	2	7.1%	0	14	16.5%	1	0.350
Controllable Lifestyle	41	100.0%	-	167	94.9%	1	0.214	13	100.0%	0	88	96.7%	0	1.000	28	100.0%	0	79	92.9%	1	0.334
Family Time	38	92.7%	-	163	92.6%	1	1.000	11	84.6%	0	84	92.3%	0	0.313	27	96.4%	0	79	92.9%	1	0.679
Work Limited hours	37	92.5%	1	159	90.9%	2	0.514	11	84.6%	0	82	90.1%	0	0.625	26	96.3%	1	77	91.7%	2	0.677
Long Working Hours	6	15.4%	2	6	3.4%	1	0.010	3	25.0%	1	3	3.3%	1	0.021	3	11.1%	1	3	3.5%	0	0.147
High Salary	13	32.5%	1	51	29.0%	1	0.703	4	33.3%	1	27	29.7%	0	0.750	9	32.1%	0	24	28.2%	1	0.811
Private Practice	12	30.0%	1	65	37.1%	2	0.467	6	46.2%	0	31	34.1%	0	0.537	6	22.2%	1	34	40.5%	2	0.108
Reasonable Ratio Income to Lifestyle	37	92.5%	1	130	74.3%	2	0.011	12	100.0%	1	67	75.3%	2	0.064	25	89.3%	0	63	73.3%	0	0.118
Academic Opportunities	6	15.0%	1	11	6.3%	3	0.098	2	16.7%	1	4	4.4%	1	0.146	4	14.3%	0	7	8.3%	2	0.463
									0.0%												
Prestigious (Population)	3	7.5%	1	13	7.4%	1	1.000	1	8.3%	1	5	5.5%	0	0.534	2	7.1%	0	8	9.4%	1	1.000
Prestigious (Colleagues)	0	0.0%	1	4	2.3%	2	1.000	0	0.0%	1	3	3.4%	2	1.000	0	0.0%	0	1	1.2%	0	1.000
Popular Specialty	16	40.0%	1	52	29.5%	1	0.257	3	25.0%	1	24	26.7%	1	1.000	13	46.4%	0	28	32.6%	0	0.257
Specialty in Crisis	10	25.6%	2	24	13.8%	3	0.089	4	33.3%	1	13	14.8%	3	0.119	6	22.2%	1	11	12.8%	0	0.233

The students not interested in FM, agreed with those interested in FM, that the specialty affords a controllable lifestyle and the ability to work limited hours (Table 3). However, they more often perceived FM as being a boring specialty and less often perceived it as providing a reasonable income to lifestyle ratio (Table 3). Additionally, these students rated the selection criteria, academic opportunities and a prestigious specialty, more highly than did students interested in FM (Table 2). Yet, they perceived FM as not being prestigious nor as affording academic opportunities (Table 3). Overall, the students perceived the specialty as not affording academic opportunities, with only 15% of those interested in FM reporting so.

Only 26% of students interested in FM and 14% of those not so inclined, perceived FM as being a specialty in crisis (NS, Table 3).

## Discussion

This study’s major aim was to utilize a marketing research model to provide medical educators, department chairs and residency program directors with objective information on Israeli medical students' perception of FM's working conditions, remuneration and clinical activities. This study, thus, examined how these perceptions align with the students’ specialty selection criteria [10, 15]. These data are important since it is extremely difficult to attract potential "buyers" (students) to a "product" (specialty) they consider unattractive. In a market environment, products that are unattractive are often modified to meet consumers’ expectations and/or are subjected to novel marketing strategies [15].

The alignment of the selection criteria of FM oriented students with their perceptions of FM is to be expected within our marketing model wherein a product that meets the selection criteria of the consumer is eminently salable. The real challenge for vendors is to entice additional consumers, i.e. those not interested in FM, to purchase their product. In marketing parlance, the vendor wishes to increase "market share" [16]. Although these consumers agreed with those interested in FM that it affords a controllable lifestyle and the ability to work limited hours, they more often perceived FM as being a boring specialty. They also less often perceived it as providing a reasonable income to lifestyle ratio, likely because among their important selection criteria was interest in private practice with its potential to boost income. Additionally, non-FM inclined students rated academic opportunities and a prestigious specialty as important selection criteria more often than did those interested in FM. However, their perceptions of FM was that it is not prestigious nor does it afford academic opportunities. Therefore, among these non-FM inclined students there is a disconnect between the specialty selection criteria and their perceptions of FM.

When there is a disconnect between a consumer's purchasing wishes (i.e. selection criteria) and his/her perception of a product, vendors must act to either dispel or modify these perceptions and/or modify the product to better meet the consumer's expectations. For example, the perception that FM is a boring specialty, likely because it has few procedures and little "action" (selection criteria rated highly by non-FM inclined students), can possibly be dispelled by cultivating more mentor-mentee relationships between students and FM faculty and by exposing students to FM practices where procedures are routinely performed.

Israeli FM was also perceived by 71% of the students not interested in FM as not providing high salaries and by 94% as not providing academic opportunities. However, the former perception does not reflect the true state of affairs, especially following significant salary increases provided to FM specialists in the 2011 union contract between the Israel Medical Association (IMA) and the Ministry of Health [17]. Therefore, this study demonstrates the need to include wages levels and provide comparisons with other specialties when marketing FM to students. Such salary information is especially important when marketing FM to male students who placed greater importance on private practice as a specialty selection criterion than did female students. The perception that FM suffers from a lack of academic opportunities should also be dispelled, especially, since Israeli medical schools increasingly use ambulatory sites for medical student clerkships. This issue also needs to be addressed at the medical school and health system levels with emphasis placed on training, recruiting and retaining academic FM physicians. The students' perceptions that FM suffers from extremely low prestige both in the eyes of their colleagues and the public points to the need for better public relations on part of both FM professional societies and leaders of the health maintenance organizations, medical schools and healthcare system. These leaders should publicly recognize and communicate the centrality and vital importance of FM to the healthcare system, especially to medical students [18].

Another marketing point to be made when marketing FM to non-FM inclined students is that FM was seen by most Israeli students as not being a stressful specialty, having a reasonable ratio of income to lifestyle and providing a controllable lifestyle. These positive perceptions are attributable to the working conditions in Israel, where primary care providers are completely exempt from nighttime and weekend working commitments. These findings contrast with realities operative in many other countries, where FM is unpopular among medical students due to uncontrollable lifestyles and low salaries leading to weak relationships between lifestyle and income [19–23].

The choice of FM as a career depends on multiple factors including medical school curricula providing and encouraging exposure to FM, the healthcare system's support of primary care, legislative initiatives designed to encourage FM careers and market forces that improve the remuneration and working conditions for FM specialists [24].

To successfully market FM to medical students requires country-specific approaches since student preferences and perception may differ between countries. For example, in the United States many students are attracted to high-paying specialties because of loan debts that need repayment [25]. In a meta-analysis on the determinants of primary care specialty choice in the US, students' characteristics found to be associated with primary care career choice were: being female, older, and married; having a broad undergraduate background; having non-physician parents; having relatively low income expectations; and having less interest in prestige, high technology and surgery [26]. In Slovenia, Ster et al. [27] found that students whose intended career choice was FM had more positive attitudes towards family physicians’ competences and towards characteristics of FM and primary care than other students. The FM inclined students described versatile and challenging work, comprehensive doctor–patient relationships, opportunities to meet people of different age groups and various backgrounds, long-term patient relationships and a well-paid job as the most attractive features of FM. In Germany, Deutsch et al. [28] surveyed how physicians choose or reject a career in FM concluding that the ways to draw more graduates to FM are attractive working conditions, academic endeavors and the external presentation of the specialty. The observations made in these articles are very similar to those found in the present study. However, unlike other countries where the shortage of FM specialists is well recognized by medical students, less than a quarter of the Israeli students considered FM to be suffering from a workforce crisis. This latter observation provides a challenge to Israeli FM leaders when marketing their specialty.

### Implications for the medical education system

In Israel FM clerkships are part of the curricula of all five medical schools. Some Israeli schools briefly expose students during their first year whereas others delay contact with FM until the final year. The latter was the case at the Hebrew University, Hadassah School of Medicine during the study period where there was a 2-week FM rotation during the final year. Therefore, it is possible to compare our findings to some European countries where FM is not well represented in the undergraduate curriculum [29–33]. Brekke et al [29] found that 19% of the medical schools from 12 different European countries had no or a very brief GP/FM exposure. Pfarrwaller et al [34] found in their literature review, that a wide primary care exposure before and during clinical training were the only interventions that were consistently associated with attracting significant numbers of students to primary care. Isolated modules or clerkships were not as effective. Similarly, in the UK, a focus group study found that early, high-quality, ongoing and authentic clinical exposure during medical school promotes general practice and combats negative stereotyping [35]. Therefore, an important tactic in FM recruitment efforts is early positive exposure in the medical school curriculum [36, 37]. This is the approach taken by the European Academy of Teachers in General Practice (EURACT), which is launching efforts to improve exposure to primary care in all medical schools [29]. Other proposed interventions to increase the proportion of medical students choosing FM include medical school admission policies favoring students interested in primary care, giving preference to students with characteristics likely to predict a future primary care career and changing the composition of admission committees to increase the number of primary care members [26]. These steps were taken in the US by the Generalist Physician Initiative and the Interdisciplinary Generalist Curriculum Project [38, 39].

### Implications for the health care system

Israel is facing a looming shortage of primary care physicians, especially in its peripheral areas. Contributing to his looming shortage is the impending retirement of many primary care practitioners, especially, those elderly physicians who emigrated from Russia in the early 1990's. In a 2015 report 44% of the community primary care physicians were not specialists in any field, with many being immigrant physicians [40]. Only 32% were FM specialists and 12% were internal medicine specialists with the remainder having specialty certification in a variety of specialties [40]. In 2015, 38% of FM specialists were older than 55 years [41]. The impact of these impending retirements is compounded by a maldistribution of physicians among the various specialties including insufficient young physicians specializing in FM. The recent increase in medical school class size, opening of a fifth medical school and increases in newly issued medical licenses increases the pool of potential recruits to FM residencies [42]. Therefore, this is an opportune time to apply the lessons of the present study. The challenge to the FM leadership is twofold. Firstly, to ensure that students expressing interest in FM medicine actually enter FM residencies and are not enticed to switched to other primary care specialties, such as internal medicine and pediatrics, which are in the same interest cluster as family practice [13]. These two specialties often tempt students to pursue subspecialization leading to their loss as primary care providers. The other challenge is recruiting some non-FM inclined students to FM, recognizing that success might be limited given that many highly rated selection criteria indicating a surgical/procedural orientation. However, recruiting even a few non-FM inclined students would help increase the number of FM specialists.

### Limitations and strengths

A weakness is that the questionnaire did not specifically examine the major reasons that students were attracted to FM and thus there may be other factors that were operative, such as the influence of mentors and role models. The influences of mentors and role models were emphasized by Matson et al. [43] who described the "4 pillars" possibly influencing students choosing FM: 1. A pipeline promoting interest in FM to secondary school students; 2. The process of medical education (role modeling FM during medical school); 3. Practice (the interplay of learners with good FM practices); and 4. Salary (narrowing the gap between primary and specialty care). Although the response rate of 66% was high for a study of this type, it is unknown whether the students who did not answer differed from the group that did answer. The strength of this study is the marketing research approach used to explore the topic of specialty selection by medical students. This methodology allowed us to compare both sides of the selection issue, the students' selection criteria and their perceptions of FM, thus demonstrating areas that could be the focus of recruitment efforts.

## Conclusions

The present study provides marketing points to help recruit non-FM inclined students to FM, including the need to maintain favorable working conditions while providing maximal financial reward; providing ample undergraduate exposure to FM's positive aspects including the scope of practice; and upgrading the reputation of the specialty among medical students. These recommendations promise to assist the healthcare system leadership in maintaining and even strengthening primary care, a major pillar of the health care system, by attracting more students to specialize in FM.

## Abbreviation

FM: Family medicine
